# Numerical Study on the Seismic Performance of Cold-Formed Steel Shear Walls with Steel Sheathing and Gypsum Board

**DOI:** 10.3390/ma16165685

**Published:** 2023-08-18

**Authors:** Shen Liu, Ruoqiang Feng, Yuting Zhong

**Affiliations:** 1 The Key Laboratory of Concrete and Pre-Stressed Concrete Structures of the Ministry of Education, Southeast University, Nanjing 211189, China; liushen@seu.edu.cn (S.L.);; 2 School of Civil Engineering, Southeast University, Nanjing 211189, China

**Keywords:** cold-formed steel, numerical modeling, seismic performance, monotonic test, cyclic test

## Abstract

The cold-formed steel shear wall with steel sheathing has gained increasing popularity due to its excellent shear capacity. To extend the applicability of this system to multi-story residences, aside from experimental investigations on the shear walls, it is essential to conduct a comprehensive study on the seismic performance of buildings. In this paper, numerical simulations were conducted on specimens subjected to monotonic and cyclic loading. Subsequently, seismic analysis of mid-rise building models was also carried out to investigate the influence of the proposed shear wall on building seismic performance. The research findings indicate that this study’s modeling method can effectively simulate the shear performance of the proposed shear wall under monotonic and cyclic loading. In addition, the proposed shear wall significantly enhances the structural stiffness and improves the seismic performance of the structure under seismic action.

## 1. Introduction

Cold-formed steel (CFS) structures enjoy widespread use in many countries and design specifications [1]. However, with the development of urbanization, an increasing number of mid-rise CFS structures have been constructed in the 21st century. The increasing number of stories requires a larger shear and compressive capacity, and different solutions have been proposed, including the steel-sheathed CFS shear wall [2,3,4], strap-braced CFS shear wall [5,6], reinforced concrete CFS shear wall [7], and so on [8,9].

The steel-sheathed CFS shear wall system has been well applied and studied because of its higher shear performance. Balh, N. et al. [3] improved the design of steel-sheathed CFS-framed shear walls for inclusion in the North American standards, using a database of monotonic and reversed cyclic shear wall tests from research programs in Canada and the US. Yu et al. [2,10] conducted a series of tests on steel sheet sheathed CFS shear walls, considering the steel sheathing thickness, aspect ratio, framing web depth, and so on. The nominal shear strength and seismic shear strength of the CFS shear walls were established for design purposes. Steel-sheathed CFS framing dynamic tests were modeled in OpenSees by Iman Shamim et al., using Pinching 04 hysteretic material [4]. The results showed that these advanced models were able to accurately reproduce the shear strength, displacement time history, and hysteretic response of the structures. Saeed Mohebbi et al. [11] compared the performance of CFS shear walls with single- and double-sided steel sheathing. The results showed that walls using double-sided sheathings have larger energy dissipation, shear strength, and elastic stiffness compared with those of single-sided sheathed walls. With the use of sheathing on both sides of the chord, stud failure must be avoided.

In general, previous research on CFS structures has predominantly focused on experimental investigations, with relatively limited attention given to numerical simulation studies, particularly those concerning the seismic behavior of buildings. While conducting cyclic and monotonic tests on CFS structures is the most direct and authentic approach to studying seismic performance, the considerable expenditure in terms of time, human resources, and costs imposes limitations on the efficiency and quantity of research conducted. Thus, the utilization of finite element (FE) simulations, allowing for a broader scope of structural seismic performance analysis and a deeper exploration of seismic design methodologies, stands as a frequently employed technique in the present field of structural engineering.

A CFS shear wall system with gypsum board and steel sheet was proposed by Feng et al., previously, and experimental research on lateral performance was conducted [12]. The proposed system adopts a gypsum board over the steel sheathing, thus enhancing both the shear capacity and fire resistance of the shear wall. However, further investigation is needed into the numerical simulation and seismic performance of the building system. Therefore, this paper conducted a monotonic loading simulation of the proposed shear walls based on ABAQUS 6.14.4 as well as a cyclic loading simulation based on OpenSeeS 3.5.0 and verified the predictive accuracy of these FE models. Additionally, the seismic performance of the building model consisting of the proposed shear walls was analyzed under frequent and rare earthquakes, highlighting the improvement in seismic performance provided by the proposed shear wall.

## 2. Simulation of Monotonic Test

**Figure 1:**
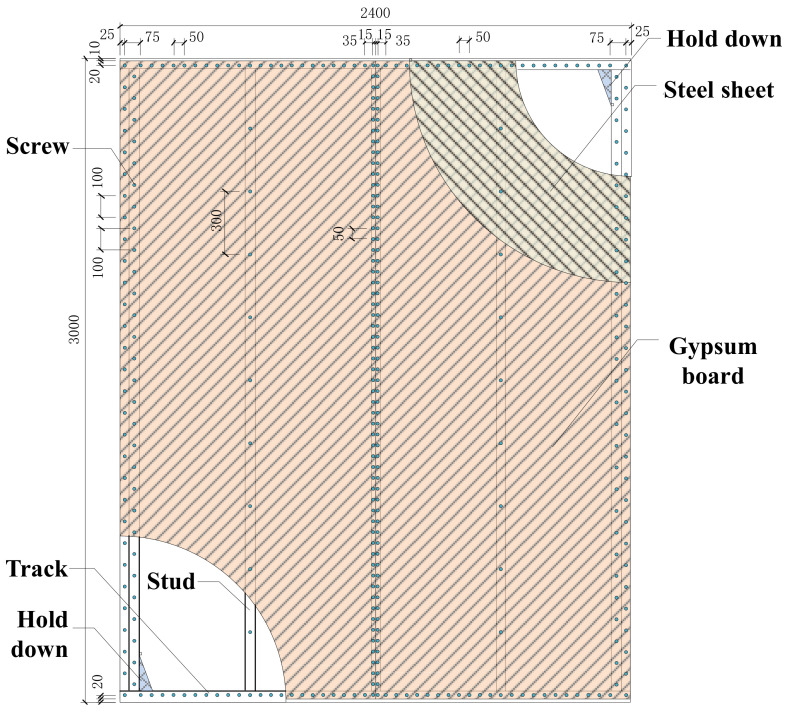
Dimensions of shear walls.

The monotonic loading tests on CFS walls with steel sheet and gypsum board were conducted previously [12]. To validate the accuracy of FE modeling methods based on the ABAQUS Standard and analyze the mechanical characteristics of the proposed shear wall, numerical simulations were performed on the two selected shear wall specimens. The dimension of the shear walls is illustrated in Figure 1, and the detailed design parameters can be found in Table 1.

### 2.1. Finite Element Modeling

#### 2.1.1. Materials and Elements

**Figure 2:**
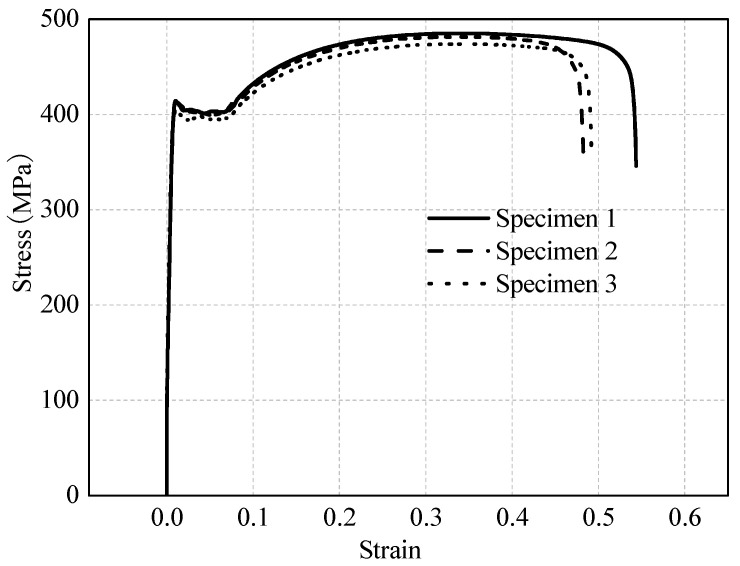
Stress-strain relationships of Q345 steel.

The CFS members of the shear wall are made of Q345 steel, and its material properties are selected based on research [12], as shown in Figure 2. The stress-strain relationship of steel is simplified as an ideal elastic-plastic model with the following parameters: elastic modulus *E* = 2.06 × 105 MPa, Poisson’s ratio *ν* = 0.3, and yield strength *f*_y_ = 395 MPa.

The mechanical properties of the gypsum board are based on the material test data provided in the research [13]. Assuming gypsum board as an isotropic ideal elastic material, the average values of the elastic modulus for both transverse and longitudinal directions are taken as 3654 MPa, and Poisson’s ratio is selected as 0.2. For a double-layer sheathing with a steel sheet as the bottom layer and a gypsum board as the face layer, the mechanical characteristics of the sheathing are primarily determined by the steel sheet. Therefore, when conducting FE modeling, this double-layer sheathing can be simplified as a single-layer steel sheathing, and the values for elastic modulus, Poisson’s ratio, and thickness can be determined by the steel sheet.

All the members, including the stud, track, and steel sheet, are simulated using a 4-node quadrilateral shell element with reduced integration and a large-strain formulation (S4R), and the overall mesh density is set to be 10 mm.

#### 2.1.2. Connections Modeling

For the CFS shear wall, the connection between the stud and track, as well as the stud and wall sheathing, is typically fastened through self-tapping screws, and the nonlinearity of the wall is mainly caused by the nonlinear behavior of the screw connections. Therefore, the simulation of screw connections is a crucial part of shear wall modeling.

**Figure 3:**
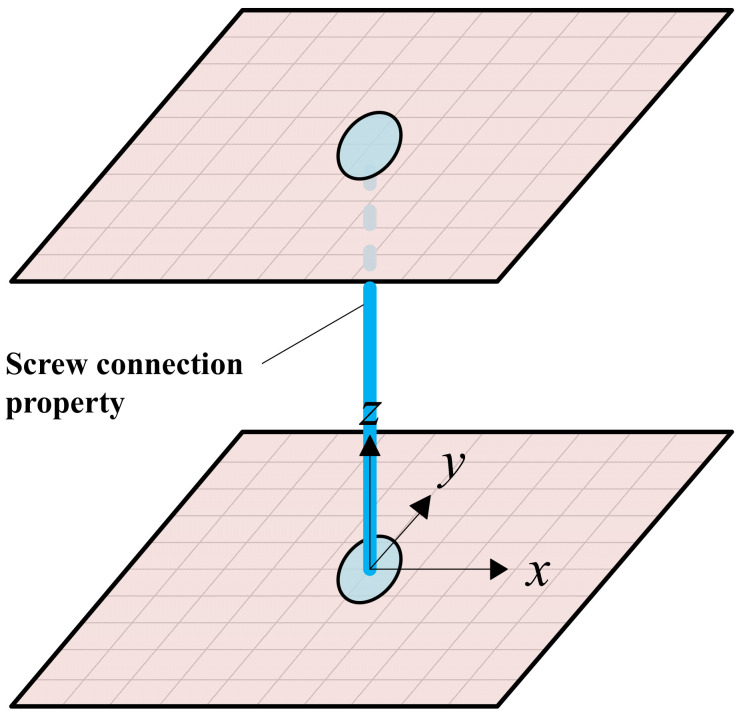
Mesh-independent fasteners in ABAQUS.

**Figure 4:**
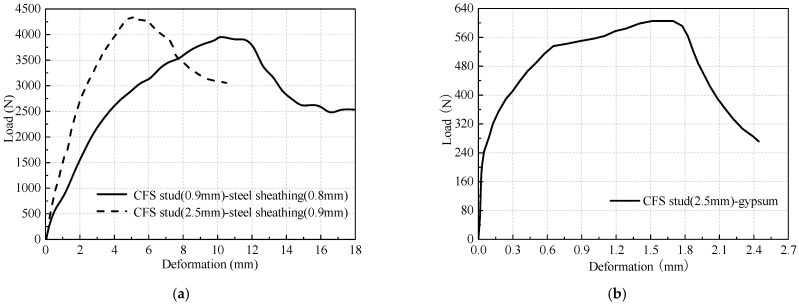
Load-deformation relationships of screwed connections: (**a**) Stud-steel sheathing connection; (**b**) Stud-gypsum connection.

In this model, the connections were modeled by mesh-independent fasteners in ABAQUS, as shown in Figure 3. Using the fastener is a convenient method to define a point-to-point connection between two or more surfaces, and these connections may be in the form of spot welds, rivets, screws, bolts, or other types of fastening mechanisms [14,15]. The properties of the fasteners are given by using the connector behavior definitions. For the stud-track connections, three translational degrees of freedom and two out-of-plane rotational degrees of freedom are restricted, except for in-plane rotational degrees of freedom. For the sheathing-stud screwed connections, the out-of-plane deformation and rotation are restrained, and the in-plane shear load-deformation behavior is obtained from the test as shown in Figure 4.

#### 2.1.3. Boundary Conditions

**Figure 5:**
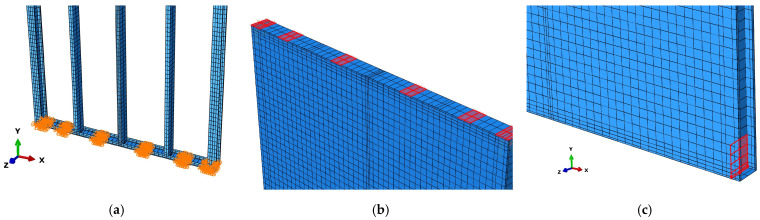
Boundary conditions of models. (**a**) Bottom track; (**b**) Top track; (**c**) Holddown.

The top and bottom tracks of the shear wall are connected to the rigid loading and boundary beams through bolts. Lateral supports are positioned on both sides of the loading beam, and holddowns are installed at the four corners of the shear wall. The translational degree of freedom in the *Z*-axis direction (out of plane) of the top track at the bolt connection is constrained, i.e., U3 = 0. Similarly, the translational degree of freedom in the *X*, *Y*, and *Z*-axis directions of the bolt connections on the bottom track is constrained, i.e., U1 = 0, U2 = 0, and U3 = 0. The holddowns are simulated using uniaxial spring elements along the *Y*-axis direction (gravity direction), with a stiffness set to 1000 N/mm. Additionally, the translational degree of freedom in the *Z*-axis direction of holddowns on the boundary studs is constrained. A reference point is established at the top of the wall, and a rigid coupling constraint is created between this point and the bolt connections of the top track, ensuring that all nodes in this area have the same translational degree of freedom as the reference point. Subsequently, a displacement is applied along the *X*-axis direction at the reference point using displacement-controlled loading. The boundary conditions are depicted in Figure 5.

### 2.2. Numerical Results

#### 2.2.1. Failure Modes

**Figure 6:**
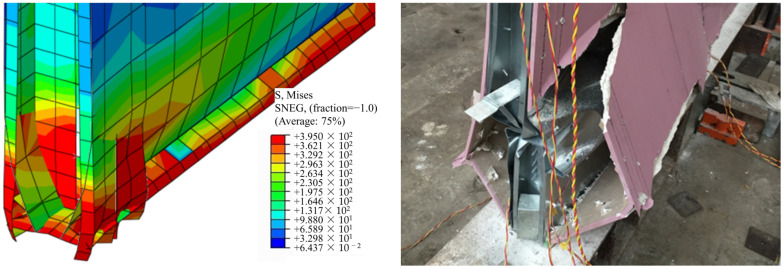
Failure comparison of specimen M1.

**Figure 7:**
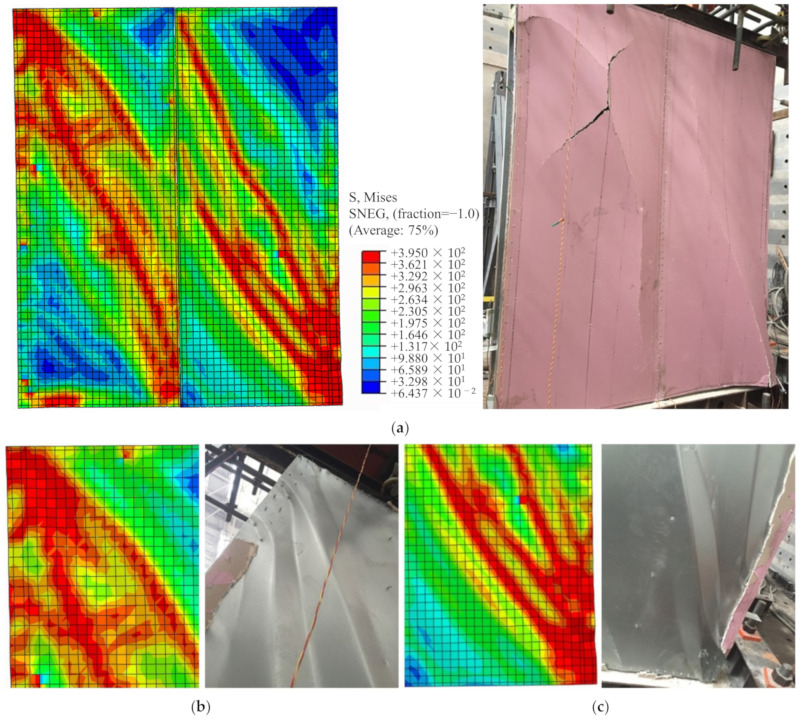
Failure comparison of specimen M2: (**a**) General view; (**b**) Upper corner; (**c**) Bottom corner.

A comparison is made between the simulated and tested failure modes of specimens M1 and M2, as shown in Figure 6 and Figure 7. It can be observed that the bottom of the boundary studs in specimen M1 experience high stress. Some areas reach a stress of 395 MPa and enter the plastic state, with noticeable local buckling and twisting failure occurring at studs. Despite the substantial tensile and compressive forces experienced by the steel sheet along the diagonal directions, the boundary studs failed before the sheet buckled. This failure mode is relatively sudden and should be avoided in the design, as the buckling of the studs would simultaneously decrease the compressive capacity of the wall. As for specimen M2, substantial tensile and compressive forces are experienced by the steel sheet along the diagonal directions, and significant bulging can be observed in the steel sheet along the diagonal directions. The stress is higher in the corner area of the wall, with some areas reaching a stress of 395 MPa and entering the plastic state. In conclusion, the numerical simulation model employed in this study effectively predicts the failure modes of the test specimens.

#### 2.2.2. Load-Displacement Relationships

**Figure 8:**
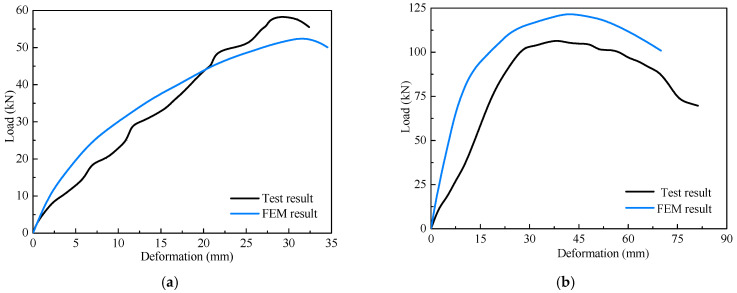
Load-displacement relationship comparison of specimens: (**a**) Specimen M1; (**b**) Specimen M2.

The simulated load-displacement curves of the shear wall are compared with the experimental results, as shown in Figure 8. In addition, the yield load *P*_y_, yield displacement Δ_y_, and shear strength *P*_s_ were calculated and summarized in Table 2. Due to experimental uncertainties, construction errors may arise, resulting in initial gaps between components. Furthermore, it is worth noting that the performance of self-tapping screw connections in the wall cannot be guaranteed due to construction quality, thus leading to potential disparities in the shear test outcomes. Hence, the initial stiffness of the specimens exceeded the test results. It is evident, according to Table 2, that the model exhibits errors within 30% when predicting yield load, peak load, and their corresponding displacements. All in all, despite visible disparities in initial stiffness, the FE method in this section can approximately predict the mechanical characteristics of the CFS shear wall with steel sheathing.

## 3. Simulation of Cyclic Test

### 3.1. Finite Element Modeling

The cyclic loading tests on CFS walls with steel sheet and gypsum board were also conducted by Feng et al. [12]. To validate the accuracy of the FE modeling method, numerical simulations were performed on the four walls mentioned in that research. The dimensions of the selected specimens are illustrated in Figure 1, and the design parameters can be found in Table 3.

#### 3.1.1. Model Simplification

The monotonic simulation using ABAQUS involves a large number of elements, resulting in a significant increase in calculation time. Therefore, OpenSees is used to simulate the hysteretic behavior of the specimens, which reduces the simulation time. The dimensions and details of the simulated shear walls are consistent with the selected specimens.

**Figure 9:**
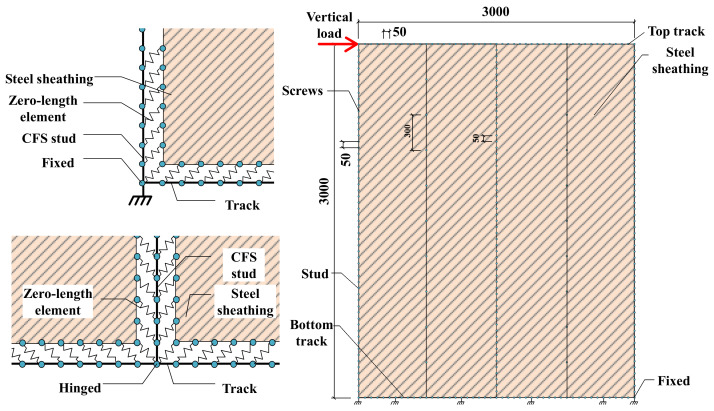
Simplified FE model.

Some simplifications are adopted during the modeling, and the typical shear wall model is shown in Figure 9. The out-of-plane constraint from the gypsum board on the steel sheet and the in-plane stiffness of the gypsum board are ignored. The influence of the gypsum board on the wall is considered through the material properties of screw connections without directly simulating the gypsum board.

Screws originally distributed in two rows with a spacing of 100 mm on the boundary studs are equivalently represented as a single row with a spacing of 50 mm, and the screw connections are simulated using zero-length elements. The connection between the middle studs and the tracks was simplified as a hinge connection. Considering the effect of the holddown, the connection between the boundary studs and tracks is simplified as a fixed connection. In addition, the bottom track is fixed at the points where it is bolted to the ground beam, and the top track is simplified as a rigid beam.

#### 3.1.2. Element Types

Displacement-based beam elements are employed for the studs and tracks. The steel sheets are modeled using ShellMITC4 elements. Zero-length elements are utilized for the screws, which connect two nodes that coincide spatially. One node is located on the stud, while the other is positioned on the steel sheet. As for the middle stud, which is connected to two steel sheets, each node on it is connected to nodes on both steel sheets simultaneously through two zero-length elements.

#### 3.1.3. Material Properties

The material properties of the models were also obtained from the research [12]. For the Displacement-based beam elements, an Elastic material model is used with an elastic modulus of 2.04 × 10^5^ MPa. As for the ShellMITC4 elements, a J2 Plasticity material model with elastic-plastic behavior is employed. The elastic modulus is 2.04 × 10^5^ MPa, the yield strength is 336.9 MPa, the ultimate strength is 447.3 MPa, and the Poisson’s ratio is 0.3.

#### 3.1.4. Screw Connections

**Figure 10:**
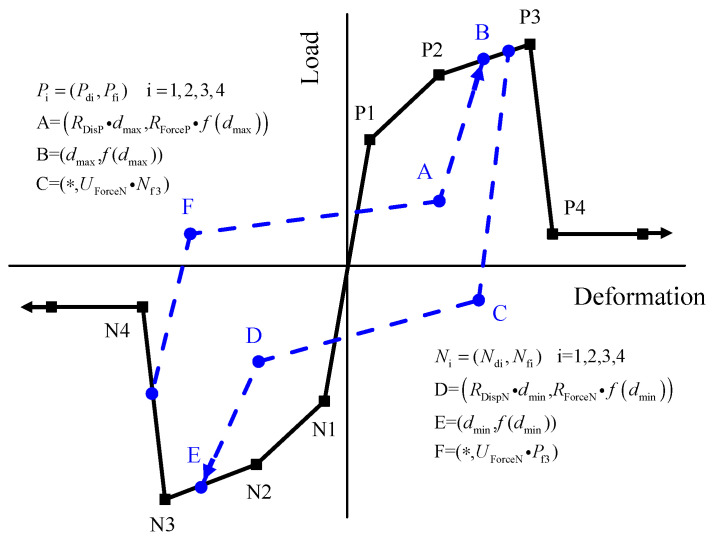
Pinching4 material model.

The zero-length elements, which are used to simulate the connections of screws in the model, utilize the Pinching4 material model [16]. The Pinching4 material incorporates stiffness degradation, strength degradation, and the pinching effect of hysteresis loops in the specimen, as shown in Figure 10. Initially, the experimentally obtained envelope curve is simplified into a multi-linear approximation. The points P1 to P4 and N1 to N4 represent the positive and negative elastic limit points, yield points, peak points, and failure points, respectively.

*P*_di_ and *P*_fi_ define displacement and force points on the positive response envelope, while *N*_di_ and *N*_fi_ define displacement and force points on the negative response envelope. (*d*_max_, *f*_max_) and (*d*_min_, *f*_min_) represent the coordinates of the maximum deformation values in the positive and negative directions, respectively. *R*_DisP_ (*R*_DisN_) is the ratio of the deformation at which reloading occurs to the maximum (minimum) historic deformation demand, while *R*_ForceP_ (*R*_ForceN_) and *U*_ForceP_ (*U*_ForceN_) are the ratios of the force at which reloading begins to the force corresponding to the maximum (minimum) historic deformation demand and the ratio of strength developed upon unloading from a negative load to the maximum (minimum) strength developed under monotonic loading, respectively. Finally, the model defines the unloading stiffness parameter *g*_Ki_, the reloading stiffness parameter *g*_Di_, and the strength degradation parameter *g*_Fi_, where i = 1, 2, 3, 4 [16].

**Figure 11:**
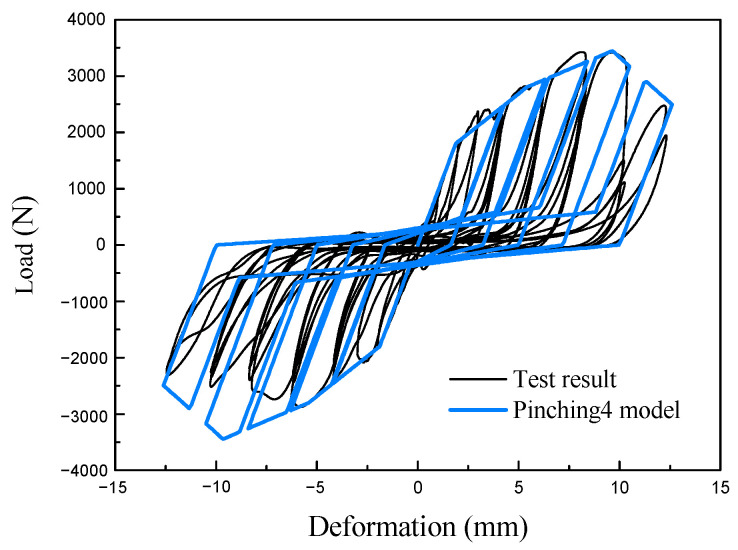
Load-deformation relationship of screwed connection and corresponding Pinching4 material model.

Due to the overall good symmetry of the experimental curves, this study adopts a symmetric simplified envelope curve. However, other parameters still have different values for the positive and negative directions, and the specific parameters are as shown in Table 4 and Table 5, and the piching4 model for the cyclic results of a 2.5 mm thick stud-0.8 mm thick sheet screwed connection is shown in Figure 11. The parameters of the screw connection with a 0.9 mm thick steel sheet are obtained by multiplying the parameters of the connections with a 0.9 mm thick steel sheet by 1.125.

#### 3.1.5. Boundary Conditions

As shown in Figure 9, the translational degrees of freedom along the *Z* direction and the rotational degrees of freedom around the *X* and *Y* directions are constrained by the nodes of the track, studs, and screwed connections. While the nodes of the steel sheets are not constrained. All the degrees of freedom of the nodes at the bolted positions of the bottom track were constrained. The boundary studs are rigidly connected to the tracks, while the other studs are hinged to the tracks. The top track is assumed to be rigid, and the loading point is set at the end of it, which is subjected to cyclic loading in displacement-controlled mode.

### 3.2. Numerical Results

**Figure 12:**
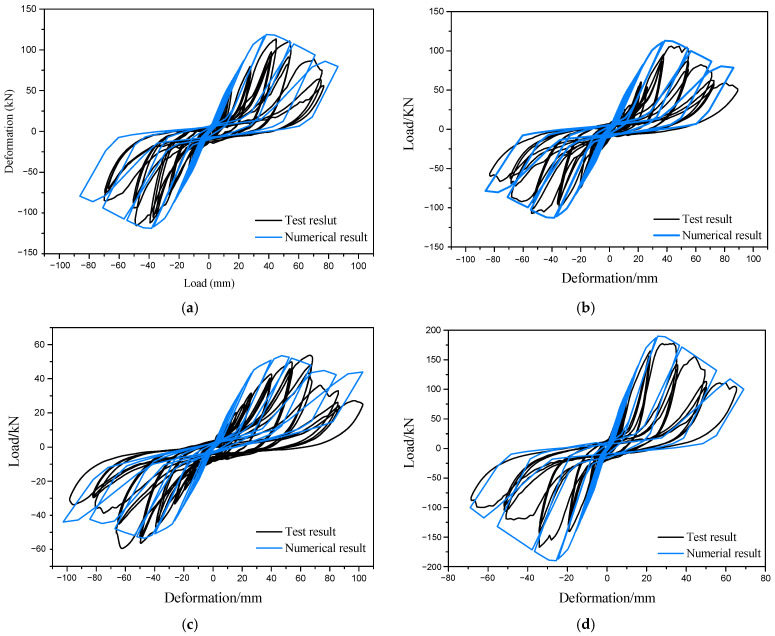
Comparison of load-displacement relationships of specimens: (**a**) Specimen C1; (**b**) Specimen C2; (**c**) Specimen C3; (**d**) Specimen C4.

The numerical results are compared with the test results, as shown in Figure 12 and Table 6. It can be observed that the hysteresis curves of the FE model generally agree with the experimental results. They exhibit pronounced “pinching” behavior and demonstrate the characteristics of strength and stiffness degradation. The ratio of yield load between the FE model and the experimental results ranges from 1.01 to 1.18, while the FE/experimental ratio of peak load ranges from 0.99 to 1.11. These small errors indicate the acceptable accuracy of the FE method.

However, except for specimen C1, the FE models of other specimens exhibit larger loading stiffness compared with the experimental results, resulting in larger deviations in both yield displacement and peak displacement. The FE/experimental ratio of yield displacement ranges from 0.67 to 1.00, and the FE/experimental ratio of peak displacement ranges from 0.71 to 0.89. This is mainly attributed to the idealized assumptions regarding the geometric model, material properties, and boundary conditions for improving computational efficiency.

## 4. Seismic Performance Mid-Rise Buildings

### 4.1. Design of Mid-Rise Buildings

**Figure 13:**
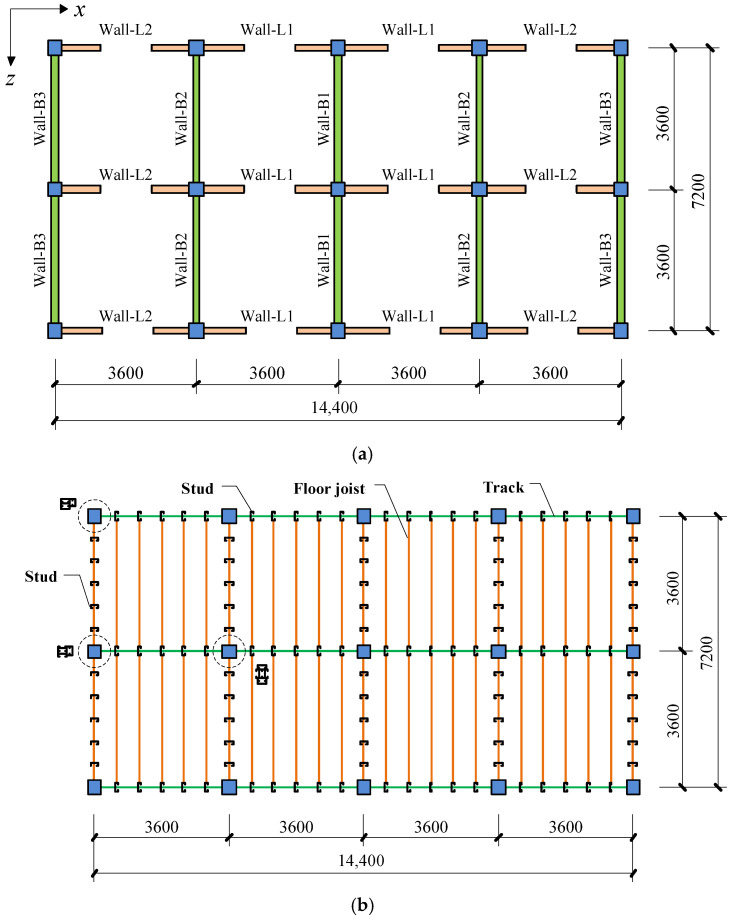
Details of the building: (**a**) Architectural floor plan; (**b**) Structural drawing.

A proposed six-story CFS building is designed and modeled using OpenSees to study the seismic performance of the proposed system. The plan dimensions are 14.4 m × 7.2 m, with a floor-to-floor height of 3 m. The architectural floor plan and structural layout are shown in Figure 13. C-Channels with dimensions of 140 × 50 × 13 × 2.5 mm (web depth × flange size × lip size × thickness) are employed for the studs, and C-Channels with dimensions of 255 × 50 × 15 × 1.5 mm are used for the joists. The stud and joist spacing is selected to be 600 mm. The floor slabs consist of 100 mm thick autoclaved lightweight concrete (ALC) panels with a 25 mm thick reinforced concrete topping. Gypsum boards are used for the ceilings. Two types of shear walls, including Wall-GC and Wall-GS, are selected for the buildings. Wall-GC is the traditional CFS wall with a 1.5 m × 1.5 m window opening. The wall panel comprises a 12 mm thick gypsum board on the inner layer and a 12 mm thick Cement Straw Board (CSB) on the outer layer. Wall-GS is the proposed CFS shear wall sheathed with a 0.8 mm thick steel sheet and 12 mm thick gypsum board on one side and a 12 mm gypsum board on the other side.

A total of eight six-story building models are established, as shown in Table 7. The main variable parameter in the models is the proportion of the proposed CFS wall Wall-GS and the traditional CFS wall Wall-GC in the building models. This analysis aims to assess the improved seismic performance of multi-story buildings by utilizing the proposed CFS shear wall with steel sheathing. According to the Chinese code (GB 50009-2012) [17], the calculated values for the floor dead load are taken as 1.69 kN/m^2^, and the live load is taken as 2.0 kN/m^2^. The roof dead load is 0.80 kN/m^2^, and the uniformly distributed live load of the roof is 0.5 kN/m^2^. The self-weight of Wall-GC is taken as 2.04 kN/m, while the self-weight of Wall-GS is 1.43 kN/m.

### 4.2. Modeling of the Mid-Rise Buildings

**Figure 14:**
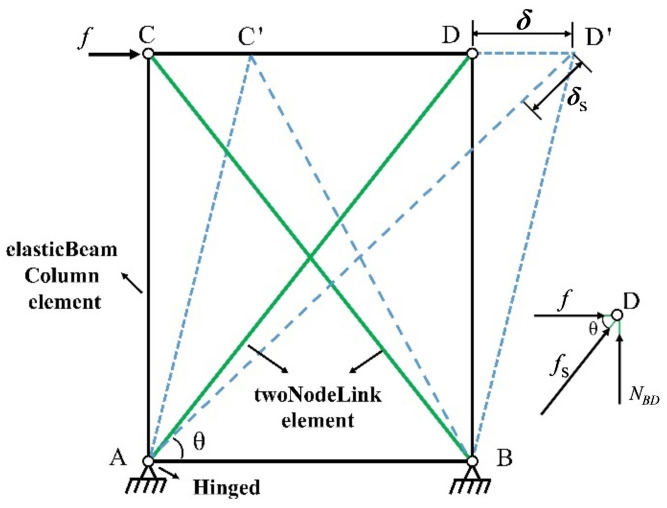
Simplified model of wall element.

The equivalent spring model can effectively simulate the hysteresis behavior of the shear wall, and this model is adopted to simplify the CFS shear walls of the models [18], as shown in Figure 14. The shear wall is simplified as hinged quadrilaterals with nonlinear spring elements (twoNodeLink elements) placed along the diagonal directions. The hinged quadrilateral consists of four rigid trusses with dimensions identical to those of the wall specimens. The nonlinear spring element has only one axial degree of freedom, and its force-displacement relationship is defined using the Pinching4 material. The relationship between spring stiffness and shear wall stiffness is as follows:

(1) $$f_{s}=f/2\cos\alpha$$

(2) $$\delta_{s}=L\prime -L=\delta \cos\alpha$$

**Figure 15:**
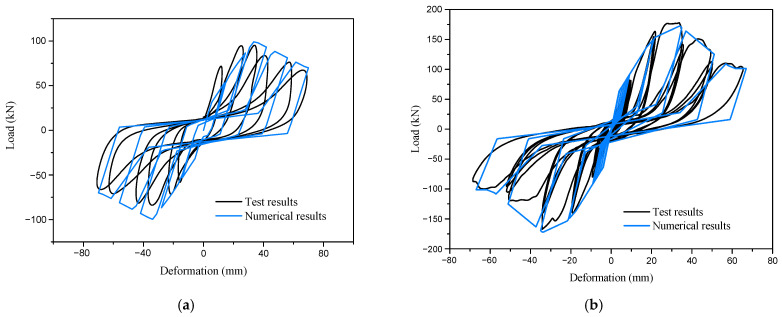
Comparison of load-displacement relationships of walls: (**a**) Wall-GC; (**b**) Wall-GS.

In which *δ* and *f* are the top horizontal displacement and the horizontal load of the wall, respectively. *δ*_s_ and *f*_s_ are the axial deformation and axial force of the nonlinear spring elements, respectively. *θ* is the angle between the spring element and the horizontal line. Two types of walls are used in the model, including Wall-GS and Wall-GC, and the corresponding Pinching4 material parameters are taken according to research [12] and research [18], respectively. The values of the calculated material parameters are given in Table 8. The comparison between the simulation and experimental results for the two walls is presented in Figure 15. It is evident that the simulation outcomes effectively approximate the load-displacement curves of the walls.

**Figure 16:**
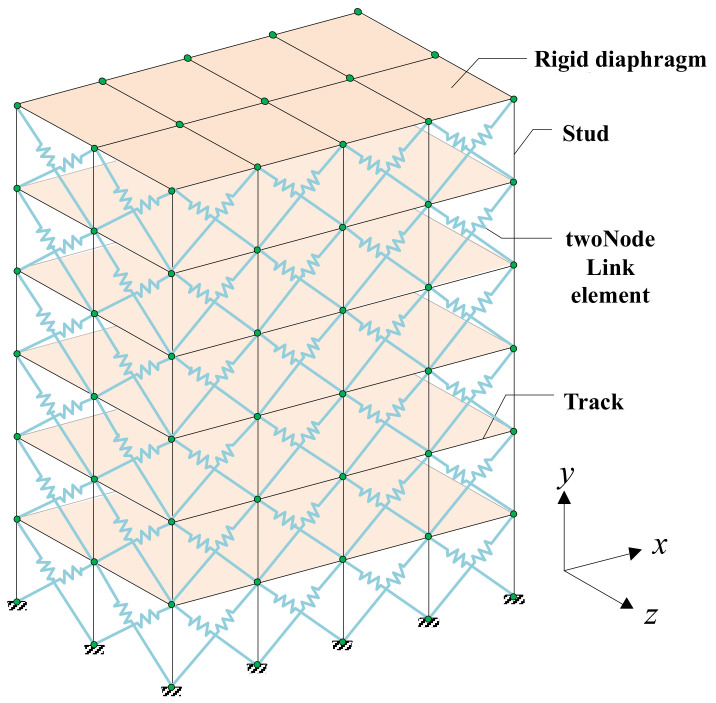
Six-story simplified building model.

The details of the model are shown in Figure 16. The elasticBeamColumn element is used to simulate the tracks and studs of the CFS walls. The twoNodeLink element is used to connect the diagonal nodes of the wall, simulating the shear contribution of the sheathings to the CFS framing. The RigidDiaphragm is used to simulate the floor slabs and roof, assuming infinite stiffness in the plane. It couples the translational and rotational degrees of freedom (Ux, Uz, and Ry) of all nodes in the floor slabs and roof in the plane. The stud-track connections and the base of the studs of the model are both set as hinges.

### 4.3. Modal Analysis

The natural periods of the first three modes of vibration for the simplified six-story building model were obtained through modal analysis and compared with the results calculated based on the ASCE/FEMA code [19] and Chinese code [20], as shown in Table 9. It can be observed that the natural periods obtained through modal analysis using OpenSees are in close agreement with the results calculated using empirical formulas based on the specifications for CFS structures [19,20]. This validates the accuracy and reliability of the proposed simplified mid-rise building models. Furthermore, as the proportion of Wall-GS increases in the model, the structure’s natural periods decrease. Compared with building model M-1, building model M-6, which utilizes only Wall-GS for the transverse walls, exhibits a reduction in the natural period of approximately 11%.

By comparing the first three modes of vibration of the building, it can be observed that the dominant mode of vibration for models M-1, M-2, and M-3 is transverse (*Z*-axis) displacement, the second mode is longitudinal (*X*-axis) displacement, and the third mode is a torsional deformation with some transverse displacement. For models M-4, M-5, and M-6, the dominant mode of vibration is longitudinal displacement; the second mode is transverse displacement; and the third mode is a torsional deformation with some longitudinal displacement. Therefore, it can be concluded that when the proportion of the Wall-GS exceeds 60% in the transverse walls, the transverse stiffness of the building structure will be greater than the longitudinal stiffness.

### 4.4. Elastic Analysis

The response spectrum method is used to calculate and analyze the structural response of buildings under frequent earthquakes. The calculation method is as follows [21]:

(3) $$F_{\mathrm{ji}}=\alpha_{j}\gamma_{j}X_{\mathrm{ji}}G_{i}\left(i=1,2,\cdots n,j=1,2,\cdots m\right)$$

(4) $$\gamma_{j}=\sum_{i=1}^{n}X_{\mathrm{ji}}G_{i}/\sum_{i=1}^{n}X_{\mathrm{ji}}^{2}G_{i}$$

In which *F*_ji_ is the characteristic value of horizontal earthquake action for the mass point i in mode j. *α*_j_ is the seismic coefficient corresponding to the natural period of mode j. *X*_ji_ is the relative horizontal displacement of the mass point i in mode j. *γ*_j_ is the participation coefficient of mode j.

After obtaining *F*_ji_, the seismic action effect can be determined. When the ratio of natural periods between adjacent modes is less than 0.85, the combined horizontal seismic action effect can be calculated using Equation (5):

(5) $$S_{\mathrm{Ek}}=\sqrt{\sum S_{j}^{2}}$$

where

*S*_Ek_ is the horizontal seismic action effect.

*S*_j_ is the horizontal seismic action effect of mode j.

The displacement at the center point of the rigid roof is taken as the roof displacement of the building. The maximum roof displacement is then calculated and recorded for the models under seismic actions with peak ground accelerations (PGA) of 0.035 g, 0.07 g, and 0.14 g, as shown in Table 10.

It can be observed that as the proportion of Wall-GS increases, maximum roof displacement gradually decreases. This indicates that the use of CFS shear walls with steel sheathing in the building enhances the lateral stiffness and improves the seismic performance of the structure. Comparing the maximum roof displacement of the models under different seismic actions with different PGAs, it is evident that the use of Wall-GS results in a more significant improvement in the seismic performance as the PGA increases. For instance, the maximum roof displacement of model M-6 decreases by 43.3% and 35.1% compared with building M-1, which has no Wall-GS, under the seismic actions with PGAs of 0.035 g and 0.14 g, respectively.

**Figure 17:**
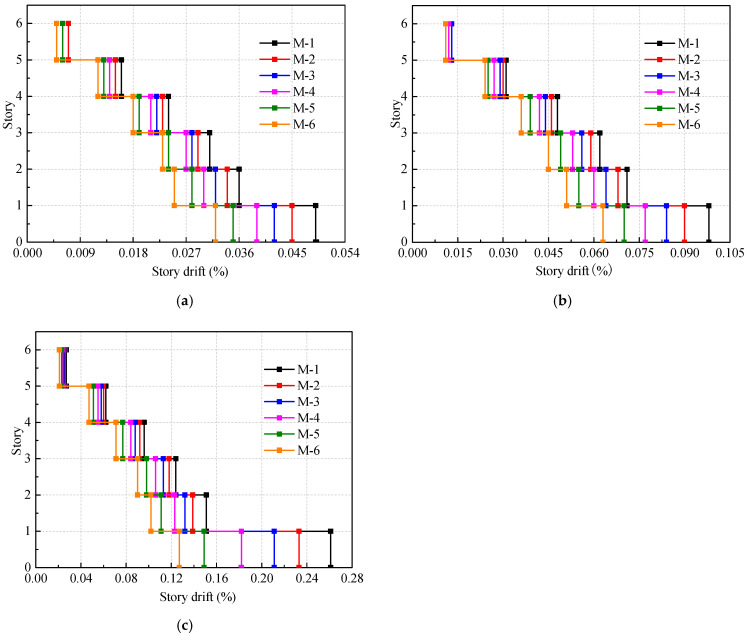
Maximum story drift ratio under frequent earthquakes: (**a**) PGA: 0.035 g; (**b**) PGA: 0.07 g; (**c**) PGA: 0.14 g.

The maximum story drift ratios of the model under the seismic actions with the PGA of 0.035 g, 0.07 g, and 0.14 g are also obtained, as shown in Figure 17. The elastic limit for the story drift ratio generated by the characteristic value of frequent earthquake action is 1/300 according to specifications JGJ 227-2011 [20]. It can be observed that the maximum story drift ratio of all the models complies with the Chinese code. As the proportion of Wall-GS in the model increases, the story drift ratio of each floor gradually decreases, indicating an increase in the overall stiffness of the building and an improvement in seismic performance.

Comparing the maximum story drift ratio of the models reveals that as the PGA increases, the use of the Wall-GS leads to a more significant improvement in the seismic performance of the structure. For example, the maximum story drift ratio of model M-6 decreases by 33.9%, 36.0%, and 50.6%, respectively, compared with building M-1 under the seismic actions with PGAs of 0.035 g, 0.07 g, and 0.14 g. This suggests that the proposed steel-sheathed CFS shear wall exhibits better enhancement in structural seismic performance under frequent earthquakes with larger amplitudes.

### 4.5. Elastoplastic Analysis

**Figure 18:**
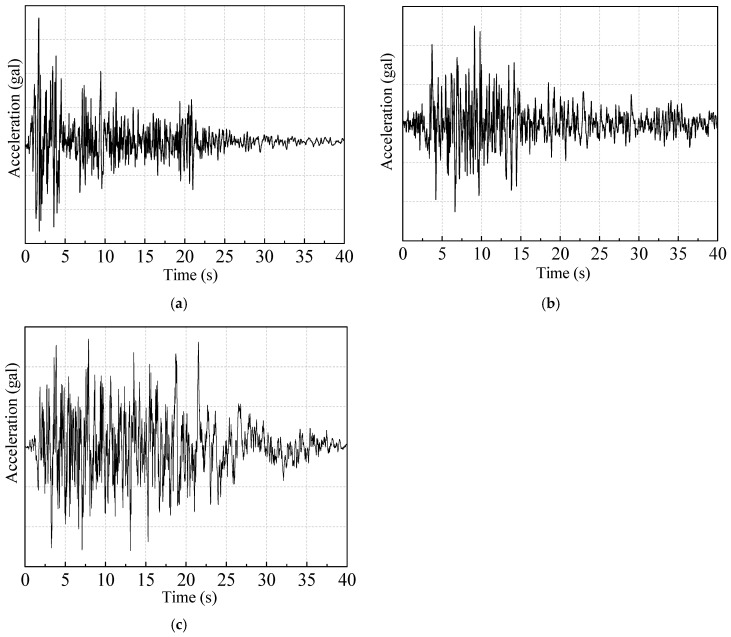
Acceleration time history curves of seismic waves: (**a**) El Centro; (**b**) TAFT; (**c**) artificial.

The elastoplastic time-history analysis is performed on the building models subjected to lateral seismic loading, investigating their response under rare earthquake actions with GPAs of 0.22 g, 0.40 g, and 0.62 g. Ground motion records El Centro waves, TAFT waves, and artificial waves are selected as the input ground acceleration in the transverse direction (*Z*-axis). The acceleration time-history curves of the seismic waves are shown in Figure 18. The maximum roof displacement is obtained, as shown in Table 11. It can be observed that as the proportion of Wall-GS in the models increases, the maximum roof displacement gradually decreases. The maximum roof displacement of model M-6 under rare earthquake action with GPAs of 0.22 g, 0.40 g, and 0.62 g decreases by an average of 32.4%, 28.8%, and 24.1%, respectively, compared with model M-1.

**Figure 19:**
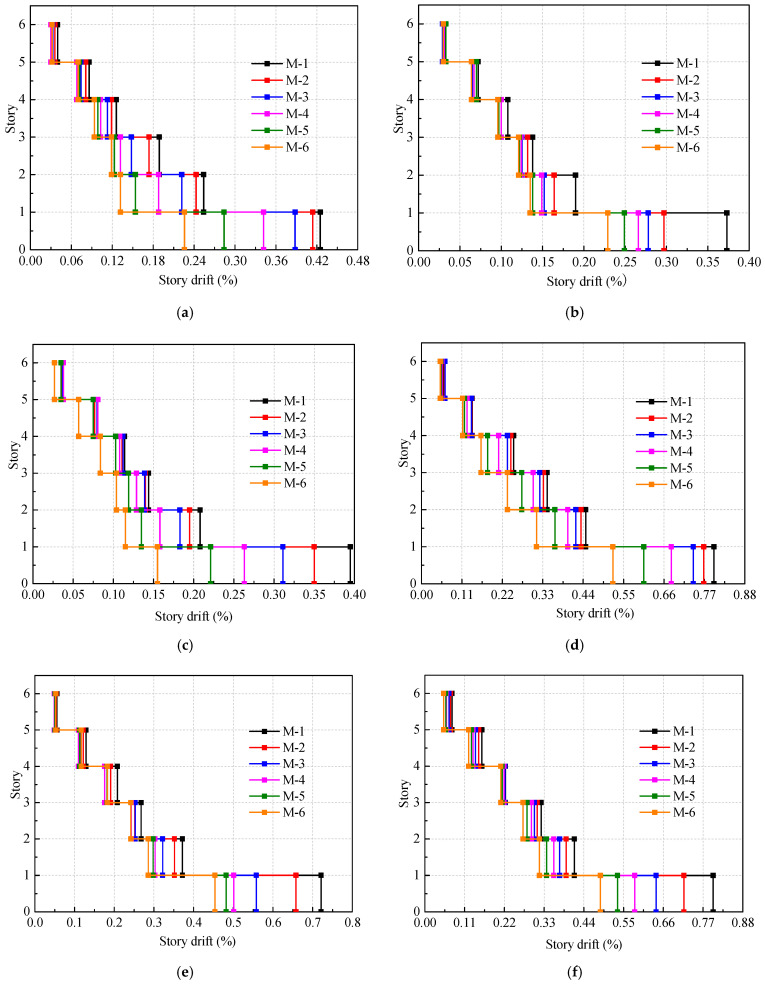

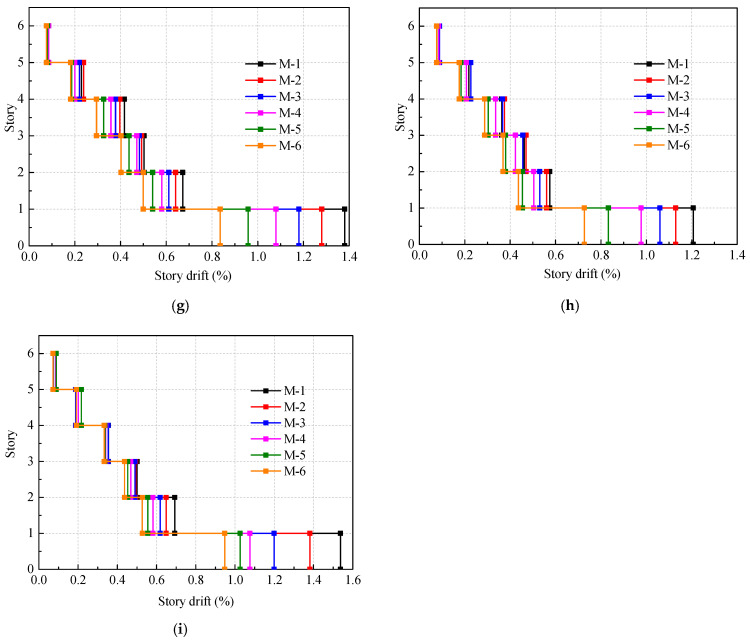
Maximum story drift ratio under rare earthquakes: (**a**) PGA: 0.22 g, El Centro; (**b**) PGA: 0.22 g, TAFT; (**c**) PGA: 0.22 g, Artificial; (**d**) PGA: 0.40 g, El Centro; (**e**) PGA: 0.40 g, TAFT; (**f**) PGA: 0.40 g, Artificial; (**g**) PGA: 0.62 g, El Centro; (**h**) PGA: 0.62 g, TAFT; (**i**) PGA: 0.62 g, Artificial.

In addition, the maximum story drift ratios of the models are also obtained, as shown in Figure 19. In accordance with the Chinese code (GB 50011-2010) [21], the story drift ratio for multi-story CFS structures under rare earthquakes is limited to 1/50. Meanwhile, the American code (ASCE/FEMA 356) [19] sets the story drift ratio limit for CFS structures at 3/100. It can be illustrated from Figure 19 that the maximum story drift ratios of all the models under rare earthquakes satisfy the limits specified by these two specifications.

Furthermore, it can also be observed from Figure 19 that as the proportion of Wall-GS in the model increases, the maximum story drift ratio of the structure gradually decreases. The maximum story drift ratio of model M-6 under rare earthquake actions with GPAs of 0.22 g, 0.40 g, and 0.62 g decreases by an average of 48.1%, 37.5%, and 37.6%, respectively, compared with model M-1.

By considering both the maximum roof displacement and the maximum story drift ratio of the models under rare earthquakes, it can be concluded that as the proportion of the CFS shear walls with steel sheathing increases, the stiffness of the structure increases, leading to improved seismic performance, which aligns with the conclusions drawn from the elastic analysis under frequent earthquakes.

## 5. Conclusions

In this paper, the simulation of the CFS shear wall with steel sheathing and gypsum board under monotonic and cyclic loading was conducted, and the accuracy of these models was verified by the experimental results. Additionally, the seismic performance of the building model consisting of the proposed shear walls was analyzed under frequent and rare earthquakes to investigate the influence of the proposed shear wall on building seismic performance. The numerical results have led to the following conclusions:

1. The CFS shear wall with steel sheathing and gypsum board exhibits two primary failure modes, namely, boundary stud buckling failure and steel sheet failure. The FE method employed in this study can effectively simulate the shear performance of the proposed shear wall under monotonic loading and predict the failure modes of specimens.
2. The modeling method used in this study, including model simplification and screw connection modeling using Pinching4 material, could effectively simulate the hysteresis behavior of the proposed shear wall under cyclic loading.
3. Through verification of natural vibration periods, the equivalent spring model demonstrates good capability for simulating the seismic performance of the proposed shear wall system.
4. Through a comparison of the maximum roof displacement and maximum story drift ratio between models subjected to frequent and rare earthquakes, it can be concluded that the proposed shear wall significantly enhances the structural stiffness and improves the seismic performance of the structure under seismic action.

## Figures and Tables

**Table 1:** Design parameters of shear walls in monotonic test [12].

Name	Dimensions	Sheathing		Stud Thickness	Steel Sheet Thickness
		Side 1	Side 2		
M1	3.0 m × 2.4 m	steel sheet + gypsum	steel sheet + gypsum	0.9 mm	0.8 mm
M2	3.0 m × 2.4 m	steel sheet + gypsum	gypsum	2.5 mm	0.9 mm

**Table 2:** Comparison of test and FE results.

Name		Yield Point		Peak Point		*P*_s_ (kN/m)
		*P*_y_ (kN)	Δ_y_ (mm)	*P*_max_ (kN)	Δ_max_ (mm)	
M1	Test results	101.9	28.1	106.6	38.9	44.4
	FE results	102.8	19.6	121.6	40.7	50.7
	FE/test	1.01	0.70	1.14	1.05	1.14
M2	Test results	53.5	26.2	58.4	29.1	24.3
	FE results	44.6	20.7	52.8	31.7	22.0
	FE/test	0.83	0.79	0.90	1.09	0.91

**Table 3:** Design parameters of specimens in cyclic test [12].

Name	Dimensions	Sheathing		Stud Thickness	Steel Sheet Thickness
		Side 1	Side 2		
C1	3.0 m × 2.4 m	steel sheet + gypsum	gypsum	2.5 mm	0.9 mm
C2	3.0 m × 2.4 m	steel sheet + gypsum	gypsum	2.5 mm	0.8 mm
C3	3.0 m × 2.4 m	steel sheet + gypsum	gypsum	2.5 mm	0.8 mm
C4	3.0 m × 3.6 m	steel sheet + gypsum	gypsum	2.5 mm	0.8 mm

**Table 4:** Pinching4 material parameters of envelope curve and unloading/reloading.

Name	Force (kN)				Displacement (mm)				Unloading and Reloading Parameters		
	*P*_f1_	*P*_f2_	*P*_f3_	*P*_f4_	*P*_d1_	*P*_d2_	*P*_d3_	*P*_d4_	*R*_DisP_	*R*_ForceP_	*U*_ForceP_
C2.5S0.8	2.576	3.296	3.856	1.578	2.40	3.60	4.80	12.00	0.6	0.2	0
C2.5S0.9	2.898	3.708	4.338	1.775	2.40	3.60	4.80	12.00	0.6	0.2	0

Note: C2.5S0.8-GS represents the 2.5 mm thick CFS stud-0.8 mm thick sheet connection with gypsum boards on both sides.

**Table 5:** Unloading stiffness, reloading stiffness, and strength degradation parameters of Pinching4 material.

Name	*g*_Ki_			*g*_Di_			*g*_Fi_	
	*g*_K1,2_	*g*_K3,4_	*g*_KLim_	*g*_D1,2_	*g*_D3,4_	*g*_DLim_	*g*_F1,2,3,4_	*g*_FLim_
C2.5S0.8	0.5	0.2	0.3	0.08	1.5	0.25.	0.0	0.0
C2.5S0.9	0.5	0.2	0.3	0.08	1.5	0.25.	0.0	0.0

**Table 6:** Comparison of test and FE results.

Name		Yield Point		Peak Point		*P*_s_ (kN/m)
		*P*_y_ (kN)	Δ_y_ (mm)	*P*_max_ (kN)	Δ_max_ (mm)	
C1	Test results	101.9	38.9	113.4	44.9	47.7
	FE results	107.3	31.3	118.9	38.4	49.5
	FE/test	1.05	0.80	1.05	0.86	1.04
C2	Test results	100.6	40.5	105.8	43.2	44.4
	FE results	102.1	31.5	112.8	38.4	47.0
	FE/test	1.01	0.78	1.07	0.89	1.06
C3	Test results	47.9	49.5	53.9	66.7	47.3
	FE results	48.2	33.1	53.6	47.1	44.7
	FE/test	1.01	0.67	0.99	0.71	0.95
C4	Test results	165.8	22.3	178.1	33.8	47.9
	FE results	171.7	20.9	190.2	25.6	52.8
	FE/test	1.04	0.94	1.07	0.76	1.10

**Table 7:** Wall types of building models.

No	Wall-B1	Wall-B2	Wall-B3	Wall-L1	Wall-L2	The Proportion of Wall-GS (%)		
						*Z* Direction	*X* Direction	All
M-1	Wall-GC	Wall-GC	Wall-GC	Wall-GC	Wall-GC	0	0	0
M-2	Wall-GS	Wall-GC	Wall-GC	Wall-GC	Wall-GC	20	0	9.1
M-3	Wall-GC	Wall-GS	Wall-GC	Wall-GC	Wall-GC	40	0	18.2
M-4	Wall-GS	Wall-GC	Wall-GS	Wall-GC	Wall-GC	60	0	27.3
M-5	Wall-GC	Wall-GS	Wall-GS	Wall-GC	Wall-GC	80	0	36.4
M-6	Wall-GS	Wall-GS	Wall-GS	Wall-GC	Wall-GC	100	0	45.5

**Table 8:** Pinching4 material parameters for Wall-GC and Wall-GS.

Name	Dimensions	Shear Capacity		Pinching4 Material Parameters							
		*P*_max_	Δ_max_	*P*_f1_	*P*_f2_	*P*_f3_	*P*_f4_	*P*_d1_	*P*_d2_	*P*_d3_	*P*_d4_
Wall-GC	3.0 m × 3.6 m	90.7	35.3	23.4	56.7	78.1	54.7	3.2	13.9	21.8	44.2
Wall-GS	3.0 m × 3.6 m	178.1	33.8	41.4	89.7	115.9	67.3	2.9	13.6	25.9	47.4

**Table 9:** Natural periods of the first three modes of vibration for models.

Name	Wall-GS Proportion (%)			Simulated Natural Periods			Specified Natural Periods
	*X*-Axis	*Y*-Axis	All	T_1_	T_2_	T_3_	
M-1	0	0	0	0.473	0.432	0.157	ASCE/FEMA Code [19] T = 0.05 H^3/4^ = 0.437 Chinese Code [20] T = 0.02 H~0.03 H = 0.36~0.54
M-2	20	0	9.1	0.443	0.430	0.149	
M-3	40	0	18.2	0.428	0.417	0.143	
M-4	60	0	27.3	0.426	0.395	0.142	
M-5	80	0	36.4	0.424	0.376	0.141	
M-6	100	0	45.5	0.421	0.359	0.140	

Note: H is the height of the structure.

**Table 10:** Maximum roof displacement under frequent earthquakes (mm).

Name	Wall-GS Proportion (%)			Peak Ground Acceleration		
	*X*-Axis	*Y*-Axis	All	0.035 g	0.07 g	0.14 g
M-1	0	0	0	4.85	9.79	21.18
M-2	20	0	9.1	4.61	9.23	19.83
M-3	40	0	18.2	4.38	8.75	18.69
M-4	60	0	27.3	3.17	8.20	17.22
M-5	80	0	36.4	2.99	6.21	15.29
M-6	100	0	45.5	2.75	5.71	13.74

**Table 11:** Maximum roof displacement under rare earthquakes (mm).

Name	Wall-GS (%)		PGA: 0.22 g			PGA: 0.40 g			PGA: 0.62 g		
	*X*	*Y*	El Centro	TAFT	Artificial	El Centro	TAFT	Artificial	El Centro	TAFT	Artificial
M-1	0	0	33.14	25.17	28.54	59.98	49.82	56.67	91.86	80.08	97.97
M-2	20	0	31.32	23.04	26.80	57.10	46.58	53.76	88.63	79.83	91.33
M-3	40	0	29.04	21.79	25.85	55.25	41.83	51.12	85.69	77.34	86.66
M-4	60	0	25.38	21.47	23.24	51.20	39.78	48.64	80.51	71.83	81.07
M-5	80	0	21.03	19.91	20.41	45.54	38.71	46.16	73.41	65.56	78.16
M-6	100	0	18.71	20.03	19.05	38.97	34.90	44.54	67.73	62.05	74.99

## Data Availability

No new data were created or analyzed in this study. Data sharing is not applicable to this article.

## References

[1] Schafer B.W. (2011). Cold-formed steel structures around the world: A review of recent advances in applications, analysis and design. Steel Constr..

[2] Yu C., Chen Y.J. (2011). Detailing recommendations for 1.83 m wide cold-formed steel shear walls with steel sheathing. J. Constr. Steel Res..

[3] Balh N., DaBreo J., Ong-Tone C., El-Saloussy K., Yu C., Rogers C.A. (2014). Design of steel sheathed cold-formed steel framed shear walls. Thin-Walled Struct..

[4] Shamim I., Rogers C.A. (2013). Steel sheathed/CFS framed shear walls under dynamic loading: Numerical modelling and calibration. Thin-Walled Struct..

[5] Fiorino L., Terracciano M.T., Landolfo R. (2016). Experimental investigation of seismic behaviour of low dissipative CFS strap-braced stud walls. J. Constr. Steel Res..

[6] Zeynalian M., Ronagh H.R. (2012). A numerical study on seismic performance of strap-braced cold-formed steel shear walls. Thin-Walled Struct..

[7] Xu Z.F., Chen Z.F., Osman B.H., Yang S.H. (2018). Seismic performance of high-strength lightweight foamed concrete-filled cold-formed steel shear walls. J. Constr. Steel Res..

[8] Dao T.N., van de Lindt J.W. (2013). Seismic Performance of an Innovative Light-Frame Cold-Formed Steel Frame for Midrise Construction. J. Struct. Eng..

[9] Derveni F., Gerasimidis S., Schafer B.W., Peterman K.D. (2021). High-Fidelity Finite Element Modeling of Wood-Sheathed Cold-Formed Steel Shear Walls. J. Struct. Eng..

[10] Yu C. (2010). Shear resistance of cold-formed steel framed shear walls with 0.686 mm, 0.762 mm, and 0.838 mm steel sheet sheathing. Eng. Struct..

[11] Mohebbi S., Mirghaderi R., Farahbod F., Sabbagh A.B. (2015). Experimental work on single and double-sided steel sheathed cold-formed steel shear walls for seismic actions. Thin-Walled Struct..

[12] Feng R.Q., Zhu B.C., Xu P.H., Qiu Y. (2019). Seismic performance of cold-formed steel framed shear walls with steel sheathing and gypsum board. Thin-Walled Struct..

[13] Ye J.H., Feng R.Q., Chen W. (2013). Seismic Technical Manual of Cold-Formed Steel Structures in Villages and Towns.

[14] (2014). Abaqus.

[15] Niari S.E., Rafezy B., Abedi K. (2015). Seismic behavior of steel sheathed cold-formed steel shear wall: Experimental investigation and numerical modeling. Thin-Walled Struct..

[16] Mazzoni S., McKenna F., Scott M.H., Fenves G.L. (2006). OpenSees command language manual. PEER Cent..

[17] (2012). Load Code for the Design of Building Structures.

[18] Wang X. (2017). Experimental and Simplified Calculation Method Research on the Lateral Performance of Mid-Rise Cold-Formed Steel Shear Walls Simulation of Shear and Compressive Performance of Cold-Formed Steel Wall. Ph.D. Thesis.

[19] (2000). Prestandard and Commentary for the Seismic Rehabilitation of Buildings.

[20] (2012). Technical Specification for Low-Rise Cold-Formed Thin-Walled Steel Buildings.

[21] (2010). Code for Seismic Design of Buildings.

